# Supplementary material for: Genome-Wide Identification of SnRK1 Catalytic α Subunit and FLZ Proteins in Glycyrrhiza inflata Bat. Highlights Their Potential Roles in Licorice Growth and Abiotic Stress Responses
Source: Int J Mol Sci. 2022 Dec 21;24(1):121. doi: 10.3390/ijms24010121 (PMC9820696; doi:10.3390/ijms24010121)
Supplement: Supplementary file 1 [file ijms-24-00121-s001.zip › ijms-2037555-supplementary.pdf]

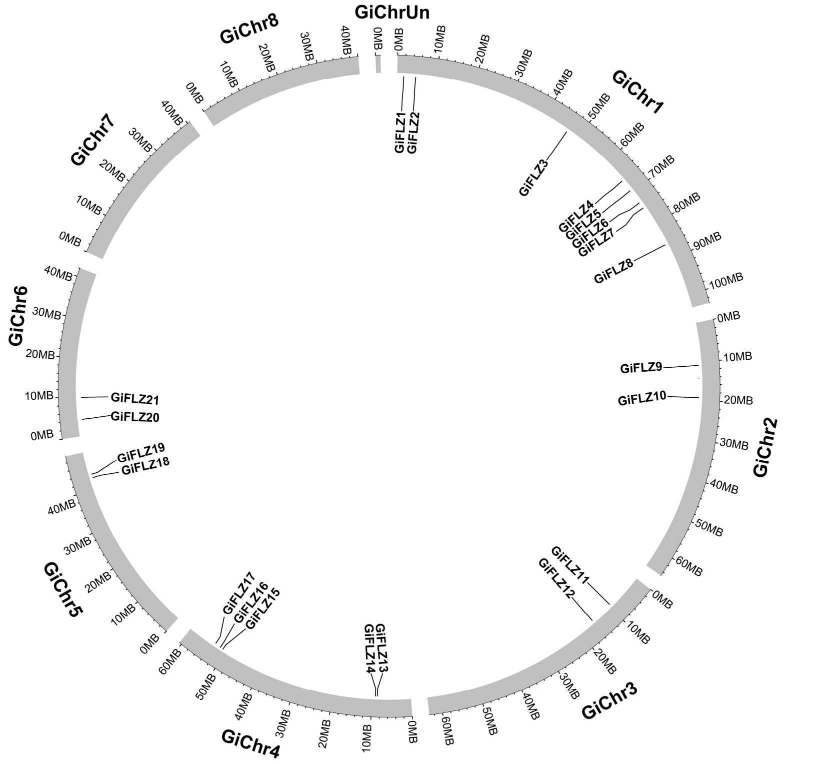

Figure S1. Chromosomal localization of *GiFLZ* genes in *G. inflata*.

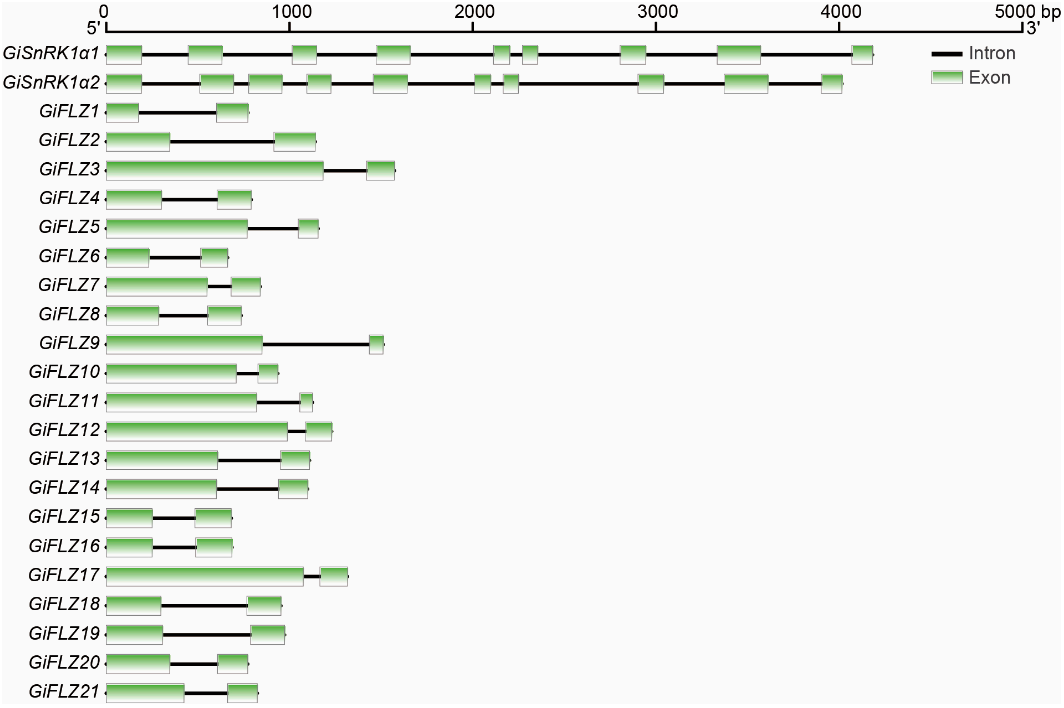

Figure S2. Exon and intron structure of *GiSnRK1α* and *GiFLZ* genes.

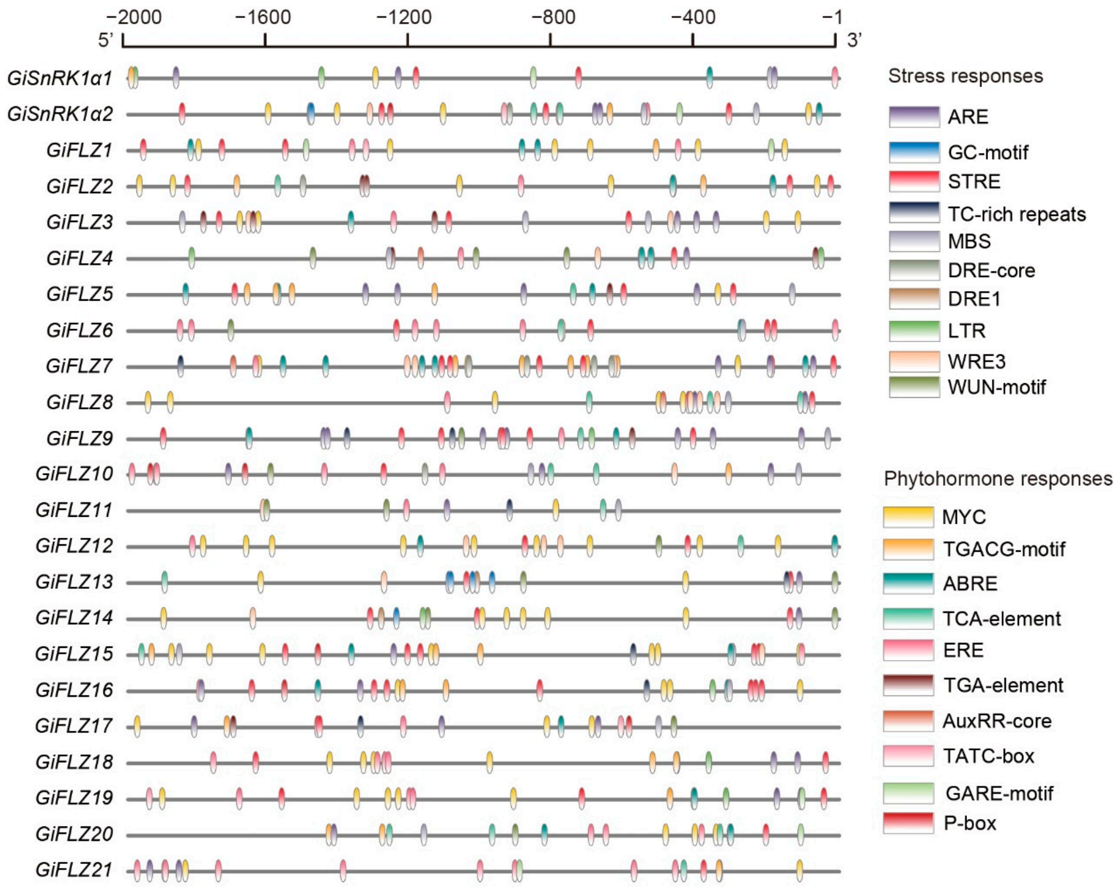

Figure S3. An overview of the distribution of cis-elements in *GiSnRK1α* and *GiFLZ* gene promoters. The promoter sequence (−2,000 bp) of the indicated genes was inferred on PlantCARE.

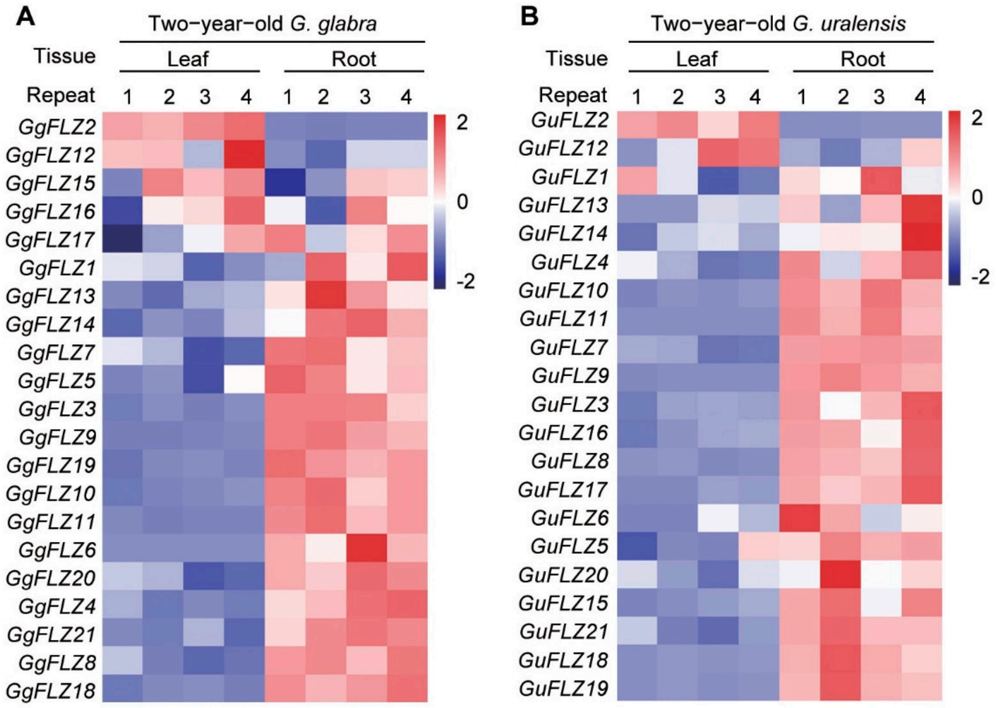

Figure S4. Expression pattern of FLZ genes in roots and leaves of 2-year-old *G. glabra* (A) and *G. uralensis* (B) plants. These expression data are normalized of FPKM and shown from low (blue) to high (red) for each gene.

Table S1. Primers used in this study.

Primer name	Sequence (5′ to 3′)	Usage
GiSnRK1α1-Y2H-F	CAGTGAATTCCACCCGATGGATGAATCAACTGGCCA	Y2H
GiSnRK1α1-Y2H-R	TATCGATGCCCACCCCTAGAGAACACGAAGCTGC	
GiFLZ2-Y2H-F	CAGTGAATTCCACCCG ATGCTACCAAGCTTCATGAG	
GiFLZ2-Y2H-R	TATCGATGCCCACCC TTATATCCGGTTGATAATTT	
GiFLZ3-Y2H-F	CAGTGAATTCCACCCG ATGCTGAGGAAGAGGAGCAG	
GiFLZ3-Y2H-R	TATCGATGCCCACCC CTATGTGTCTGTAGGAATGC	
GiFLZ4-Y2H-F	CAGTGAATTCCACCCG ATGGCTGGGTCTACCAAAAG	
GiFLZ4-Y2H-R	TATCGATGCCCACCC CTAAGCAGCAGCAACTGTTC	
GiFLZ9-Y2H-F	CAGTGAATTCCACCCG ATGGCAGAAGATTCTGGTTG	
GiFLZ9-Y2H-R	TATCGATGCCCACCC TCATATTTGGTCAATCCCAC	
GiFLZ10-Y2H-F	CAGTGAATTCCACCCG ATGGGTGATAATAGCTCCCA	
GiFLZ10-Y2H-R	TATCGATGCCCACCC TTAAACTTCTGCTCCATCTA	
GiFLZ14-Y2H-F	CAGTGAATTCCACCCG ATGATGTTGGGAAAGAGATC	
GiFLZ14-Y2H-R	TATCGATGCCCACCC CTAAAGGGCAACAATCCCAG	
GiFLZ15/16-Y2H-F	CAGTGAATTCCACCCG ATGGTGGGTCTGAGTGTAGT	
GiFLZ15/16-Y2H-R	TATCGATGCCCACCC TCAGTAAGCAAAAGCACCGG	
GiFLZ17-Y2H-F	CAGTGAATTCCACCCG ATGCTGAAGAAGAAGGAACA	
GiFLZ17-Y2H-R	TATCGATGCCCACCC TTATTTGGACACAAGCAGAC	
GiFLZ18-Y2H-F	CAGTGAATTCCACCCG ATGTTGCTGGGGAAGAGACC	
GiFLZ18-Y2H-R	TATCGATGCCCACCC CTACAAGGCAATAGCCACGG	
GiFLZ21-Y2H-F	CAGTGAATTCCACCCG ATGTTGCTTGGGAAGCGTCC	
GiFLZ21-Y2H-R	TATCGATGCCCACCC TCAAGTACAAGCTGCAGCGG	
GiSnRK1α1-GFP-F	GATTAACAGGGATCCCCCATGGATGAATCAACTGGCCAT	GFP fusion
GiSnRK1α1-GFP-R	GAGACTAGTGGTACCCCCGAGAACACGAAGCTGCGAAAG	
GiFLZ2-GFP-F	CTGATTAACAGGGATCCCCC ATGCTACCAAGCTTCATGAG	
GiFLZ2-GFP-R	TCGAGACTAGTGGTACCCCC TATCCGGTTGATAATTTGCG	
GiFLZ3-GFP-F	CTGATTAACAGGGATCCCCC ATGCTGAGGAAGAGGAGCAG	
GiFLZ3-GFP-R	TCGAGACTAGTGGTACCCCC TGTGTCTGTAGGAATGCCTG	
GiFLZ4-GFP-F	CTGATTAACAGGGATCCCCC ATGGCTGGGTCTACCAAAAG	
GiFLZ4-GFP-R	TCGAGACTAGTGGTACCCCC AGCAGCAGCAACTGTTCCTG	
GiFLZ9-GFP-F	CTGATTAACAGGGATCCCCC ATGGCAGAAGATTCTGGTTG	
GiFLZ9-GFP-R	TCGAGACTAGTGGTACCCCC TATTTGGTCAATCCCACCCT	
GiFLZ10-GFP-F	CTGATTAACAGGGATCCCCC ATGGGTGATAATAGCTCCCA	
GiFLZ10-GFP-R	TCGAGACTAGTGGTACCCCC AACTTCTGCTCCATCTAACA	
GiFLZ14-GFP-F	CTGATTAACAGGGATCCCCC ATGATGTTGGGAAAGAGATC	
GiFLZ14-GFP-R	TCGAGACTAGTGGTACCCCC AAGGGCAACAATCCCAGTTG	
GiFLZ15/16-GFP-F	CTGATTAACAGGGATCCCCC ATGGTGGGTCTGAGTGTAGT	
GiFLZ15/16-GFP-R	TCGAGACTAGTGGTACCCCC GTAAGCAAAAGCACCGGGTC	
GiFLZ17-GFP-F	CTGATTAACAGGGATCCCCC ATGCTGAAGAAGAAGGAACA	
GiFLZ17-GFP-R	TCGAGACTAGTGGTACCCCC TTTGGACACAAGCAGACCCA	
GiFLZ18-GFP-F	CTGATTAACAGGGATCCCCC ATGTTGCTGGGGAAGAGACC	
GiFLZ18-GFP-R	TCGAGACTAGTGGTACCCCC CAAGGCAATAGCCACGGTGG	

GiFLZ21-GFP-F	CTGATTAACAGGGATCCCCC ATGTTGCTTGGGAAGCGTCC	
GiFLZ21-GFP-R	TCGAGACTAGTGGTACCCCC AGTACAAGCTGCAGCGGTTT	
GiSnRK1α1-qPCR-F	TGGGAACAAGGCAACAGTTC	qRT-PCR
GiSnRK1α1-qPCR-R	GAATTTCCAAGGTAATGATTAT	
GiFLZ2-qPCR-F	GAACAGACAAATAATTATGG	
GiFLZ2-qPCR-R	AAGTCTCTGCATTCGAAGGT	
GiFLZ3-qPCR-F	CTCTGATGCTAATTTGGATA	
GiFLZ3-qPCR-R	TAGCATCTAGTGGAGAAGTG	
GiFLZ4-qPCR-F	CCGGGGTATTCTGGTCATAA	
GiFLZ4-qPCR-R	AACCGATGATGGGTTCCTGA	
GiFLZ9-qPCR-F	ATGGCAGAAGATTCTGGTTG	
GiFLZ9-qPCR-R	GCTCATCATGGTTTCAGAAC	
GiFLZ10-qPCR-F	CAGAATTACGCTAGAAGTAC	
GiFLZ10-qPCR-R	ATAGCTTCGGTCCCTAATGG	
GiFLZ13/14-qPCR-F	AGACCATCACTAAGGTTTAC	
GiFLZ13/14-qPCR-R	TGTGTTTCTTCTGATAACAC	
GiFLZ17-qPCR-F	CTGATACATCAAGTCTAAGA	
GiFLZ17-qPCR-R	GTCTGCAATCAAGAGGAGAT	
GiFLZ18/19-qPCR-F	CCACGGAGGTTGTTGACAAC	
GiFLZ18/19-qPCR-R	CGGTGCTGCTGCTGTTAATA	
GiFLZ21-qPCR-F	CACGCAGATGCAGCAGACAA	
GiFLZ21-qPCR-R	GCATGATGTGGATCTTTACT	
GiCS-qPCR-F	GCTCAGCCGTTGACCCAG	
GiCS-qPCR-R	CACCACCAGGAAAAGCACC	
